# Methyl 3-[(*E*)-furfuryl­idene]dithio­carbazate

**DOI:** 10.1107/S1600536808012506

**Published:** 2008-05-03

**Authors:** Shang Shan, Shan-Heng Wang, Ying-Li Xu, Pei-Jin Xie, Yu-Liang Tian

**Affiliations:** a College of Chemical Engineering and Materials Science, Zhejiang University of Technology, People’s Republic of China

## Abstract

The mol­ecule of the title Schiff base compound, C_7_H_8_N_2_OS_2_, prepared by the reaction of methyl dithio­carbazate and furfural in an ethanol solution under reflux, adopts an *E* configuration; the dithio­carbazate and furan units are located on opposite sides of the C=N double bond. The planar dithio­carbazate group is twisted slightly with respect to the furan ring, making a dihedral angle of 5.2 (1)°. Adjacent mol­ecules are linked by N—H⋯S hydrogen bonding to form a supra­molecular dimer across an inversion center.

## Related literature

For general background, see: Okabe *et al.* (1993 ▶); Shan *et al.* (2002 ▶, 2003 ▶). For a related structure, see: Chen *et al*. (2007 ▶). For the synthesis, see: Hu *et al.* (2001 ▶).
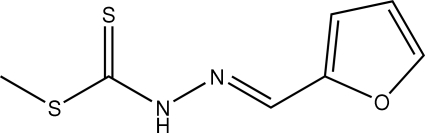


## Experimental

### 

#### Crystal data

C_7_H_8_N_2_OS_2_
*M* *_r_* = 200.27
Triclinic, 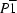

*a* = 4.0866 (8) Å
*b* = 8.8698 (12) Å
*c* = 12.8453 (15) Å
α = 93.970 (14)°
β = 91.856 (12)°
γ = 98.293 (12)°
*V* = 459.21 (12) Å^3^
*Z* = 2
Mo *K*α radiation
μ = 0.53 mm^−1^
*T* = 294 (2) K
0.34 × 0.28 × 0.20 mm

#### Data collection

Rigaku R-AXIS RAPID IP diffractometer
Absorption correction: multi-scan (*ABSCOR*; Higashi, 1995 ▶) *T*_min_ = 0.850, *T*_max_ = 0.950 (expected range = 0.804–0.899)
4733 measured reflections
1608 independent reflections
1349 reflections with *I* > 2σ(*I*)
*R*_int_ = 0.030

#### Refinement

*R*[*F* ^2^ > 2σ(*F* ^2^)] = 0.031
*wR*(*F* ^2^) = 0.096
*S* = 1.09
1608 reflections
110 parameters
H-atom parameters constrained
Δρ_max_ = 0.19 e Å^−3^
Δρ_min_ = −0.28 e Å^−3^

### 

Data collection: *PROCESS-AUTO* (Rigaku, 1998 ▶); cell refinement: *PROCESS-AUTO*; data reduction: *CrystalStructure* (Rigaku/MSC, 2002 ▶); program(s) used to solve structure: *SIR92* (Altomare *et al.*, 1993 ▶); program(s) used to refine structure: *SHELXL97* (Sheldrick, 2008 ▶); molecular graphics: *ORTEP-3 for Windows* (Farrugia, 1997 ▶); software used to prepare material for publication: *WinGX* (Farrugia, 1999 ▶).

## Supplementary Material

Crystal structure: contains datablocks I, global. DOI: 10.1107/S1600536808012506/xu2417sup1.cif

Structure factors: contains datablocks I. DOI: 10.1107/S1600536808012506/xu2417Isup2.hkl

Additional supplementary materials:  crystallographic information; 3D view; checkCIF report

## Figures and Tables

**Table 1:** Hydrogen-bond geometry (Å, °)

*D*—H⋯*A*	*D*—H	H⋯*A*	*D*⋯*A*	*D*—H⋯*A*
N1—H1⋯S1^i^	0.86	2.65	3.4892 (17)	165

Symmetry code: (i) 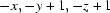.

**Fig. 1.**
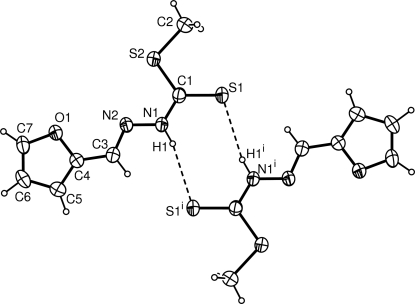
The molecular structure of the title compound with 40% probability displacement ellipsoids (arbitrary spheres for H atoms) [symmetry code: (i) -x, 1 - y, 1 - z].

## supplementary crystallographic information

### Comment

Since some hydrazone derivatives have shown the potential bioactivity as DNA-damaging or mutagenic agents (Okabe *et al.*, 1993), a lots of new hydrazone compounds has been synthesized in our laboratory (Shan *et al.*, 2002, 2003). As part of the ongoing investigation on hydrazone, we present here the crystal structure of the title compound.

The molecular structure is shown in Fig. 1. The N2═C3 bond distance of 1.284 (2) Å clearly indicates the double bond character for the Schiff base compound. The molecule adopts an E configuration, the carbazate and furan moieties located on the opposite positions of the N2═C3 bond; similar to that found in a related structure (Chen *et al.*, 2007). The dithiocarbazate moiety is well co-planar, the maximum atomic deviation being 0.037 (1) Å (S2), and the dithiocarbazate mean plane is slightly twisted with respect to the furan plane by a smaller dihedral angle of 5.2 (1)°. This shows the whole molecule is nearly co-planar.

Inter-molecular N—H···S hydrogen bonding links adjacent molecules to form the centro-symmetric supra-molecular dimmer (Fig. 1 and Table 1).

### Experimental

Methyl dithiocarbazate was synthesized in the manner reported previously (Hu *et al.*, 2001). Methyl dithiocarbazate (1.24 g, 10 mmol) and furfural (0.96 g, 10 mmol) were dissolved in ethanol (10 ml) and refluxed for 4 h. Yellow crystalline product appeared after cooling to room temperature. They were separated and washed with cold water. Single crystals of the title compound were obtained by recrystallization from an ethanol solution.

### Refinement

Methyl H atoms were placed in calculated positions with C—H = 0.96 Å and torsion angle was refined to fit electron density, *U*_iso_(H) = 1.5*U*_eq_(C). Other H atoms were placed in calculated positions with C—H = 0.93 and N—H = 0.86 Å, and refined in the riding mode, *U*_iso_(H) = 1.2*U*_eq_(C,N).

### Crystal data

**Table:**

C_7_H_8_N_2_OS_2_	*Z* = 2
*M**_r_* = 200.27	*F*_000_ = 208
Triclinic, *P*1	*D*_x_ = 1.448 Mg m^−^^3^
Hall symbol: -P 1	Melting point = 414–416 K
*a* = 4.0866 (8) Å	Mo *K*α radiation λ = 0.71073 Å
*b* = 8.8698 (12) Å	Cell parameters from 2276 reflections
*c* = 12.8453 (15) Å	θ = 2.0–25.0º
α = 93.970 (14)º	µ = 0.53 mm^−^^1^
β = 91.856 (12)º	*T* = 294 (2) K
γ = 98.293 (12)º	Prism, yellow
*V* = 459.21 (12) Å^3^	0.34 × 0.28 × 0.20 mm

### Data collection

**Table:**

Rigaku R-AXIS RAPID IP diffractometer	1608 independent reflections
Radiation source: fine-focus sealed tube	1349 reflections with *I* > 2σ(*I*)
Monochromator: graphite	*R*_int_ = 0.030
Detector resolution: 10.0 pixels mm^-1^	θ_max_ = 25.2º
*T* = 294(2) K	θ_min_ = 1.6º
ω scans	*h* = −4→4
Absorption correction: multi-scan(ABSCOR; Higashi, 1995)	*k* = −10→10
*T*_min_ = 0.850, *T*_max_ = 0.950	*l* = −15→14
4733 measured reflections	

### Refinement

**Table:**

Refinement on *F*^2^	Secondary atom site location: difference Fourier map
Least-squares matrix: full	Hydrogen site location: inferred from neighbouring sites
*R*[*F*^2^ > 2σ(*F*^2^)] = 0.031	H-atom parameters constrained
*wR*(*F*^2^) = 0.096	*w* = 1/[σ^2^(*F*_o_^2^) + (0.0598*P*)^2^] where *P* = (*F*_o_^2^ + 2*F*_c_^2^)/3
*S* = 1.09	(Δ/σ)_max_ = 0.001
1608 reflections	Δρ_max_ = 0.19 e Å^−^^3^
110 parameters	Δρ_min_ = −0.28 e Å^−^^3^
Primary atom site location: structure-invariant direct methods	Extinction correction: none

### Special details

**Table:**

Geometry. All e.s.d.'s (except the e.s.d. in the dihedral angle between two l.s. planes) are estimated using the full covariance matrix. The cell e.s.d.'s are taken into account individually in the estimation of e.s.d.'s in distances, angles and torsion angles; correlations between e.s.d.'s in cell parameters are only used when they are defined by crystal symmetry. An approximate (isotropic) treatment of cell e.s.d.'s is used for estimating e.s.d.'s involving l.s. planes.
Refinement. Refinement of *F*^2^ against ALL reflections. The weighted *R*-factor *wR* and goodness of fit *S* are based on *F*^2^, conventional *R*-factors *R* are based on *F*, with *F* set to zero for negative *F*^2^. The threshold expression of *F*^2^ > σ(*F*^2^) is used only for calculating *R*-factors(gt) *etc*. and is not relevant to the choice of reflections for refinement. *R*-factors based on *F*^2^ are statistically about twice as large as those based on *F*, and *R*- factors based on ALL data will be even larger.

### Fractional atomic coordinates and isotropic or equivalent isotropic displacement parameters (Å^2^)

**Table:**

	*x*	*y*	*z*	*U*_iso_*/*U*_eq_	
S1	0.20815 (13)	0.27619 (5)	0.50863 (4)	0.0516 (2)	
S2	0.38692 (13)	0.20142 (5)	0.28541 (4)	0.0506 (2)	
N1	0.1859 (4)	0.45265 (16)	0.35473 (12)	0.0464 (4)	
H1	0.1098	0.5149	0.3988	0.056*	
N2	0.2353 (4)	0.49265 (16)	0.25394 (11)	0.0449 (4)	
O1	0.3206 (4)	0.60245 (14)	0.05809 (10)	0.0553 (4)	
C1	0.2537 (4)	0.3197 (2)	0.38518 (14)	0.0407 (4)	
C2	0.4665 (5)	0.0393 (2)	0.35393 (17)	0.0557 (5)	
H2A	0.6311	0.0720	0.4090	0.084*	
H2B	0.5448	−0.0343	0.3062	0.084*	
H2C	0.2659	−0.0063	0.3831	0.084*	
C3	0.1483 (5)	0.6216 (2)	0.23454 (16)	0.0502 (5)	
H3	0.0577	0.6772	0.2875	0.060*	
C4	0.1847 (5)	0.6835 (2)	0.13501 (16)	0.0481 (5)	
C5	0.1076 (7)	0.8131 (3)	0.09881 (19)	0.0685 (6)	
H5	0.0139	0.8886	0.1360	0.082*	
C6	0.1961 (6)	0.8126 (3)	−0.00661 (18)	0.0673 (6)	
H6	0.1705	0.8873	−0.0523	0.081*	
C7	0.3227 (6)	0.6849 (3)	−0.02770 (17)	0.0608 (6)	
H7	0.4018	0.6557	−0.0920	0.073*	

### Atomic displacement parameters (Å^2^)

**Table:**

	*U*^11^	*U*^22^	*U*^33^	*U*^12^	*U*^13^	*U*^23^
S1	0.0714 (4)	0.0502 (3)	0.0359 (3)	0.0125 (2)	0.0107 (2)	0.0105 (2)
S2	0.0694 (4)	0.0482 (3)	0.0378 (3)	0.0171 (2)	0.0114 (2)	0.0072 (2)
N1	0.0681 (10)	0.0401 (8)	0.0326 (9)	0.0104 (7)	0.0108 (7)	0.0056 (7)
N2	0.0614 (10)	0.0393 (8)	0.0352 (9)	0.0078 (7)	0.0060 (7)	0.0077 (7)
O1	0.0837 (10)	0.0443 (7)	0.0418 (8)	0.0168 (7)	0.0145 (7)	0.0112 (6)
C1	0.0456 (10)	0.0405 (9)	0.0345 (10)	0.0013 (8)	0.0024 (8)	0.0026 (8)
C2	0.0701 (14)	0.0467 (11)	0.0534 (13)	0.0161 (9)	0.0056 (10)	0.0087 (9)
C3	0.0655 (13)	0.0478 (11)	0.0390 (11)	0.0114 (9)	0.0094 (9)	0.0051 (9)
C4	0.0631 (12)	0.0419 (10)	0.0419 (11)	0.0129 (9)	0.0072 (9)	0.0076 (8)
C5	0.0988 (17)	0.0596 (13)	0.0577 (14)	0.0391 (12)	0.0168 (12)	0.0165 (11)
C6	0.0947 (17)	0.0596 (13)	0.0544 (15)	0.0224 (12)	0.0054 (12)	0.0278 (11)
C7	0.0875 (16)	0.0583 (12)	0.0393 (12)	0.0115 (11)	0.0109 (10)	0.0173 (9)

### Geometric parameters (Å, °)

**Table:**

S1—C1	1.6675 (18)	C2—H2B	0.9600
S2—C1	1.7500 (19)	C2—H2C	0.9600
S2—C2	1.800 (2)	C3—C4	1.430 (3)
N1—N2	1.379 (2)	C3—H3	0.9300
N1—C1	1.331 (2)	C4—C5	1.344 (3)
N1—H1	0.8600	C5—C6	1.413 (3)
N2—C3	1.284 (2)	C5—H5	0.9300
O1—C4	1.361 (2)	C6—C7	1.327 (4)
O1—C7	1.364 (2)	C6—H6	0.9300
C2—H2A	0.9600	C7—H7	0.9300
			
C1—S2—C2	101.98 (9)	N2—C3—C4	122.79 (19)
C1—N1—N2	121.34 (16)	N2—C3—H3	118.6
C1—N1—H1	119.3	C4—C3—H3	118.6
N2—N1—H1	119.3	C5—C4—O1	109.57 (17)
C3—N2—N1	114.59 (16)	C5—C4—C3	132.1 (2)
C4—O1—C7	106.45 (15)	O1—C4—C3	118.37 (16)
N1—C1—S1	120.76 (14)	C4—C5—C6	106.8 (2)
N1—C1—S2	114.05 (13)	C4—C5—H5	126.6
S1—C1—S2	125.19 (11)	C6—C5—H5	126.6
S2—C2—H2A	109.5	C7—C6—C5	106.67 (19)
S2—C2—H2B	109.5	C7—C6—H6	126.7
H2A—C2—H2B	109.5	C5—C6—H6	126.7
S2—C2—H2C	109.5	C6—C7—O1	110.5 (2)
H2A—C2—H2C	109.5	C6—C7—H7	124.8
H2B—C2—H2C	109.5	O1—C7—H7	124.8
			
C1—N1—N2—C3	177.62 (17)	N2—C3—C4—C5	179.2 (2)
N2—N1—C1—S1	177.32 (12)	N2—C3—C4—O1	−0.7 (3)
N2—N1—C1—S2	−3.0 (2)	O1—C4—C5—C6	0.7 (3)
C2—S2—C1—N1	178.23 (14)	C3—C4—C5—C6	−179.2 (2)
C2—S2—C1—S1	−2.13 (15)	C4—C5—C6—C7	−0.5 (3)
N1—N2—C3—C4	179.09 (17)	C5—C6—C7—O1	0.1 (3)
C7—O1—C4—C5	−0.6 (2)	C4—O1—C7—C6	0.3 (3)
C7—O1—C4—C3	179.25 (18)		

### Hydrogen-bond geometry (Å, °)

**Table:**

*D*—H···*A*	*D*—H	H···*A*	*D*···*A*	*D*—H···*A*
N1—H1···S1^i^	0.86	2.65	3.4892 (17)	165

Symmetry codes: (i) −*x*, −*y*+1, −*z*+1.
